# Extractability of Rice Husk Waste Using Green Gamma Radiation for Dye Elimination in Laboratory-Scale Sorption System: Equilibrium Isotherm and Kinetic Analysis

**DOI:** 10.3390/ma16093328

**Published:** 2023-04-24

**Authors:** Zakia Alhashem, Ashraf H. Farha, Shehab A. Mansour, Maha A. Tony

**Affiliations:** 1 Department of Physics, College of Science, King Faisal University, Al-Ahsa 31982, Saudi Arabia; 2 Semiconductors Technology Laboratory, Physics Department, Faculty of Science, Ain Shams University, Cairo 11566, Egypt; 3 Advanced Materials/Solar Energy and Environmental Sustainability (AMSEES) Laboratory, Faculty of Engineering, Menoufia University, Shebin El-Kom 32511, Egypt; 4 Basic Engineering Science Department, Faculty of Engineering, Menoufia University, Shebin El-Kom 32511, Egypt

**Keywords:** γ-irradiation, Urolene Blue dye, bioadsorbent rice husk, radiation activation, isotherm model, kinetics

## Abstract

Nowadays, the use of natural materials and especially “waste” valorization has evolved and attracted the wide attention of scientists and academia. In this regard, the use of rice husk (RH) powder as a naturally abundant and cheap byproduct material is gaining superior attention. However, improving the physicochemical properties of such RH is still under research. In the current investigation, the modification of rice husk (RH) via γ-irradiation has shown to be a promising green tool to meet such a need. Clean, prepared, powdered RH samples were subjected to various γ-radiation doses, namely 5, 10, 15 and 25 kGy, and the corresponding samples were named as RH-0, RH-5, RH-10, RH-15, RH-15 and RH-25. Then, the samples were characterized via scanning electron microscopy (SEM). After irradiation, the samples showed an increase in their surface roughness upon increasing the γ-radiation up to 15 kGy. Furthermore, the sorption capacity of the irradiated RH samples was investigated for eliminating Urolene Blue (UB) dye as a model pharmaceutical effluent stream. The highest dye uptake was recorded as 14.7 mg/g, which corresponded to the RH-15. The adsorption operating parameters were also investigated for all of the studied systems and all adsorbents showed the same trend, of a superior adsorption capacity at pH 6.6 and high temperatures. Langmuir and Freundlich isotherm models were also applied for UB adsorption and an adequate fitted isotherm model was linked with Langmuir fitting. Moreover, the pseudo-second-order kinetic model provided the best fit for the adsorption data. Experimental assays confirmed that the UB dye could be successfully eradicated feasibly from the aqueous stream via a sustainable green methodology.

## 1. Introduction

Water is the most essential resource for life on Earth; however, wastewater pollution is categorized as one of the worst types of environmental pollution. Urbanization and modern industries have significant assets in wastewater since the industrial sector uses massive amounts of water, which results in huge quantities of wastewater streams [1]. The treatment of wastewater is of utmost importance as an alternative solution to water conservation on Earth [1]. Ongoing efforts have been applied to treat such massive amounts of wastewater, which have resulted from many industries, and the suitability of the applied methodology is based on the type and characteristics of the aqueous effluents [2]. There are four main categories of wastewater treatment methodologies, namely, physical, mechanical, biological and chemical methods [3]. The adsorption technique has been signified as one of the most efficient chemical methods. It is eco-friendly, cost-efficient and simple to handle especially in using naturally abundant materials [3,4,5,6]. However, such materials need further activation to become superior adsorbents.

Urolene Blue (UB) dye is a heterocyclic aromatic chemical compound that possesses a low molecular weight and high water solubility but with a toxic nature. Urolene Blue is used in the cosmetic and pharmaceutical industries. As a result of using this dye in various industries, releases through watercourses have occurred. The harmful effects of such a dye include skin diseases, diarrhea, hypertension and eye problems when swallowed or brought in contact with the human body. All these factors make the removal of it from wastewater streams a must [7,8].

Rice husk (RH) is categorized as one of the most abundant examples of lignocellulosic biomass, especially in rice-growing countries. Since RH results from the milling process of paddies, therefore, from the paddy milling process, approximately 22% of paddy weight results as RH [9]. A small amount of resultant RH is used for power generation and as fuel during the paddy milling process [10]. Meanwhile, RH has a negative impact on the environment, especially in developing countries, since RH is produced in massive amounts, which causes difficulties in its management as well as its costly disposal. As a result, RH is usually left unused after the milling process in farmlands or burned in situ in open air. The result of such a burning technique is extreme environmental damage as well as the waste of a valuable source of nutrients [10,11]. Moreover, burning RH in open air causes greenhouse gas emissions which result from the conversion of elements such as C, O and H into flammable gases and ash [11]. The resultant rice husk ash (RHA), from the burning of RH contains very high amounts of silica. The development of the silica causes severe damage to farmlands and the general areas where burning takes place. Silica causes strong environmental pollution since it is a non-biodegradable product. Given that 25% of RH weight is converted into RHA due to burning [10], 20% of RH is in the form of complex silica while RHA contains 95% amorphous silica [12]. RH chemically comprises cellulose, hemicellulose in high proportions, lignin in medium proportions, silica in small proportions and very small traces of metal oxides such as CaO, MgO, Al_2_O_3_ and Fe_2_O_3_ [9,13,14]. 

Over the past decades, RH valorization, which could be categorized as energy and non-energy applications, has received considerable attention [15]. Non-energy applications include animal feed and biochar for soil amendment, construction materials such as cement reinforcement and ceramic manufacturing [16,17], raw material for antibiotic production [18], silica extraction [19] and its derivatives for industrial applications. Meanwhile, energy production using RH includes heat and electricity production as well as fuel production such as biofuels, bio-oil and biochar [10,15,20]. In sum, RHA possesses superior adsorptive properties for pollutant elimination from aqueous effluents and has previously been applied for metallic pollutant and dye effluent removal [21,22]. The application of such “low cost” material in adsorption was previously investigated for RH in its natural form as an effective methodology for water decontamination [23,24]. However, it has rarely been applied in its modified form especially using a green activation technique such as γ-irradiation [25,26]. Furthermore, for practical industrial-scale application, it is essential to perform dynamic studies using column adsorption studies. According to previous studies [27,28,29], besides its simplicity, continuous availability and inexpensiveness to fabricate, rice husk has previously been proven to be a good substrate for the adsorption of a bed column for eliminating various pollutants.

γ-irradiation technology has demonstrated many potential advantages over other conventional processing methods. It is an eco-friendly technology and signified as a green process since it requires no need for a catalyst or additives for processing [30]. γ-irradiation possesses high-energy photons and short wavelengths, making it deeply penetrating into samples over a short amount of time [31]. Previous reports have applied such technology to improve the physical and mechanical properties of various rubber-blended composites and/or rice husk derivative fillers [30,32,33]. Moreover, γ-irradiation was used to induce the cross-linkage of a polymer backbone and polymer degradation without chemical reagents [31,34,35]. However, according to the authors’ knowledge, less reports have been published on the application of γ-irradiation on the physical and chemical characteristics of RH. Therefore, the current study aims to investigate the impact on RH by applying various γ-irradiation doses. 

For the purposes of the current investigation, the technology could be categorized as win-win by using waste RH material for UB removal while providing reclaimed water for further use. RH samples were initiated through exposure to 5, 10, 15 and 25 kGy doses of γ-irradiation and the corresponding samples were named RH-5, RH-10, RH-15 and RH-25 in addition to the control sample (RH-0). Surface modification of the samples was explored through SEM imaging. The samples were then exposed to UB adsorption, the system parameters were studied and the adsorption uptake was recorded. Isotherm models as well as kinetic models were investigated to highlight the adsorption RH/UB system. The current investigation introduces γ-irradiation as a green technique to enhance the adsorption uptake of RH waste. 

## 2. Materials and Methods

### 2.1. Adsorbent “Rice Husk” Collection and Preparation

Raw rice husk (RH) material was collected from a local rice mill in the Menoufia Governorate north of Egypt because it is widely known by rice producers. Initially, after the RH was collected, it was subjected to washing via distilled water. Prior to that, it was subjected to oven drying in an electric furnace at 60 °C for 2 h of drying time. Thereafter, with the primary material cleaned, the RH was subjected to further washing with acetone using magnetic stirring for 10 min. In order to get rid of any remaining contamination, a further washing step was conducted by using sodium hydroxide solution (0.3 M) under magnetic stirring for another 10 min. To maintain the cleaning process, the washing steps with acetone and sodium hydroxide were successively repeated. The obtained RH was then dried in an electric furnace at 60 °C for 5 h. For the object of activation and to attain a good adsorption capacity of the adsorbent material, the dried RH powder was exposed to various γ-radiation doses at 5, 10, 15 and 25 kGy. The source of radiation used was Cobalt-60 (India Gamma Chamber, Ge 4000A) at the National Center for Radiation Research and Technology (NCCRT) in Egypt. The dose rate used was 1.347 kGy/h. A Fricke reference standard dosimeter (ASTM E 1026, 2004) was used to calibrate the absorbed dose rate of cells. The irradiation process was performed under the conditions of the reference standard dosimeter (National Institute of Standards and Technology, Gaithersburg, ML, USA). The adsorbent samples were labeled as RH-0, RH-5, RH-10, RH-15, RH-15 and RH-25 according to the corresponding doses of γ-radiation, of 0, 5, 10, 15 and 25 kGy, respectively.

### 2.2. Adsorbent Characterization

The surface morphology of the non-irradiated and γ-irradiated RH samples was characterized via field-emission scanning electron microscopy (FE-SEM, Quanta FEJ20) and Fourier transform infrared measurements (FTIR). The transmittance FTIR spectra of the samples were obtained using a JASCO spectrometer model (FT/IR-4100) with a wave number ranging from 400 to 4000 cm^−1^.

The Brunauer–Emmett–Teller (BET) surface area of the samples was obtained using a surface area and pore size distribution analyzer (MICROTRAC MRB, BELSORP-miniX). The samples were then degassed at 60 °C for 2 h under nitrogen gas flow according to prior measurements.

### 2.3. Adsorption Methodology and Wastewater Analysis

**Figure 1:**
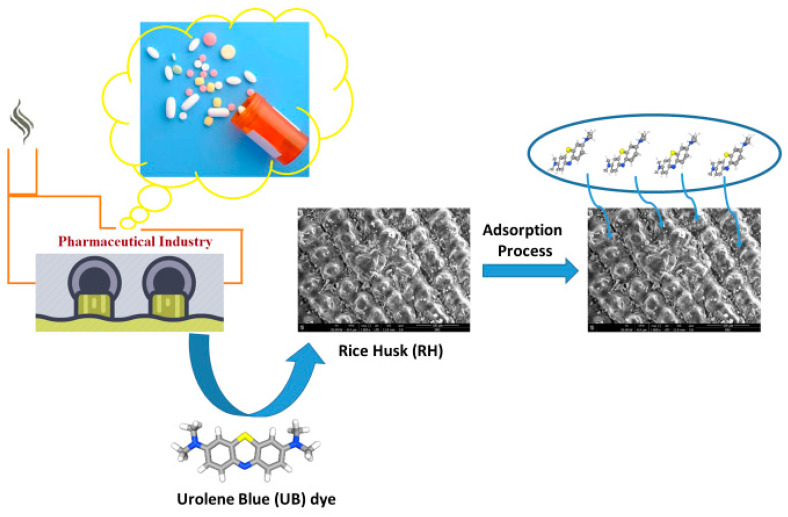
Schematic representation of the adsorption process of UB dye using RH.

In most cases, an aqueous synthetic solution of UB dye is laboratory-prepared by the initial preparation of a stock solution (1000 ppm) from the dye, which is further diluted as required, and the initial UB concentration is recorded as C_UBo_. The synthetic solution was used as a simulated wastewater effluent to imitate the pharmaceutical effluent industry discharge from a monocomponent solution using distilled water. The pH of the solution was adjusted, if required, using a digital pH meter by adding diluted solutions of H_2_SO_4_ or NaOH solutions at analytical grades. Then, an explicit dose of RH material was added to the solution to investigate its adsorption uptake. The samples were then subjected to mechanical shaking for various contact times to investigate the equilibrium isotherm time. After the isotherm time was investigated, the adsorption parameters were then investigated through the isotherm time and the remaining C_UB_ concentration was recorded to calculate the adsorption uptake. The remaining dye in the samples was recorded by spectrophotometric analysis (UV-1601, Model TCC-240A; Kyoto, Japan) according to the maximum wavelength of the UB dye. A graphical illustration of the treatment steps is represented by Figure 1.

## 3. Results and Discussion

### 3.1. Structural and Morphological Characterization of Rice Husk 

**Figure 2:**
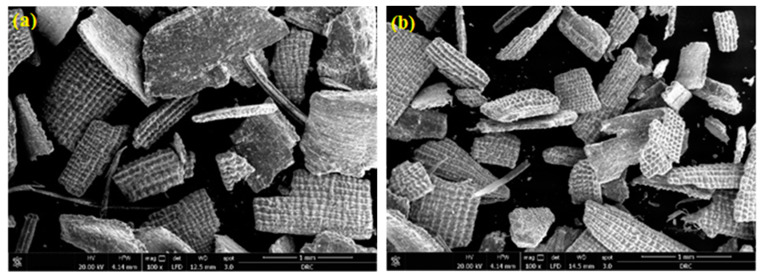
FE-SEM micrograph images under 100× magnification: (**a**) RH-0 and (**b**) RH-10.

**Figure 3:**
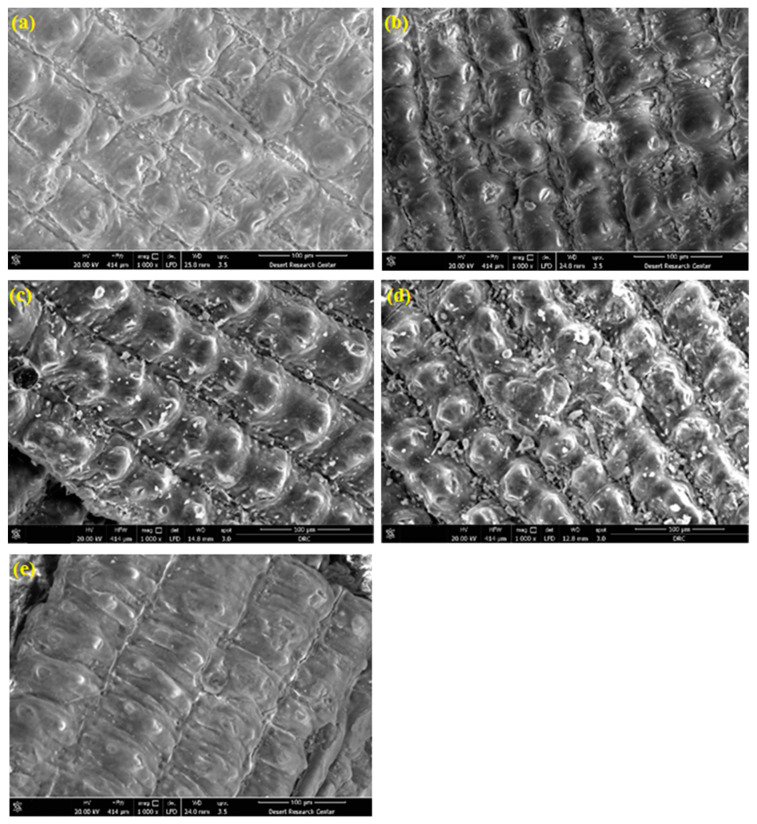
FE-SEM micrograph images with 1000× magnification for RH samples with various doses of γ-radiation: (**a**) RH-0, (**b**) RH-5, (**c**) RH-10, (**d**) RH-15 and (**e**) RH-25.

**Figure 4:**
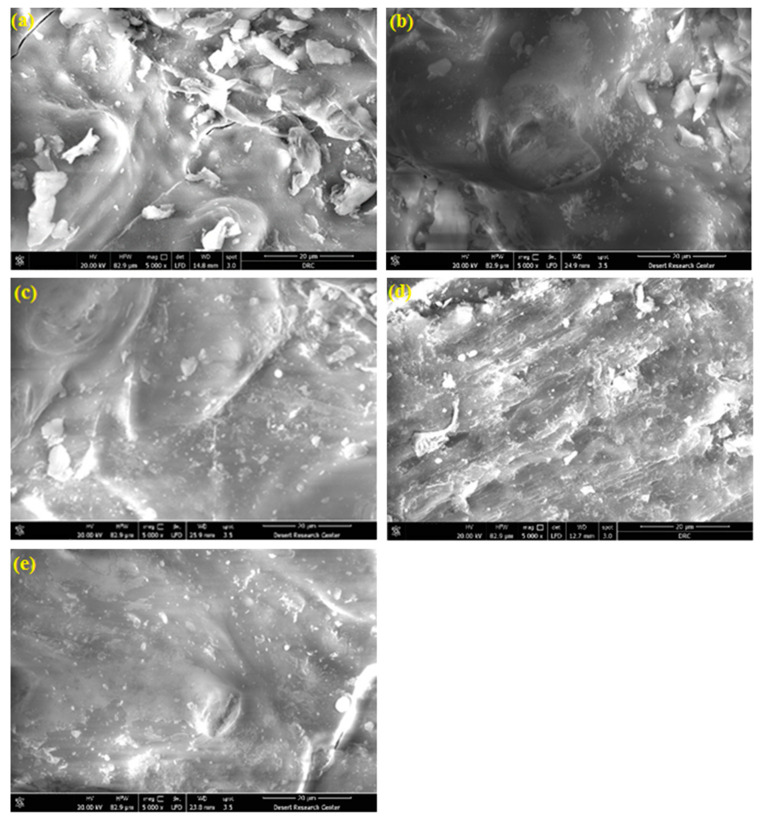
FE-SEM micrograph images with 5000× magnification for RH samples with various doses of γ-radiation: (**a**) RH-0, (**b**) RH-5, (**c**) RH-10, (**d**) RH-15 and (**e**) RH-25.

Figure 2 shows an overview of the FE-SEM micrographs for the RH-0 and RH-10 samples with (100×) magnification. The collected micrographs revealed approximately the same morphological features for samples under the selected low magnification (100×), with the same results for other samples at various irradiation doses. However, the obtained FE-SEM micrographs for all the investigated samples are shown in Figure 3 and Figure 4, which were taken under the higher magnifications of 1000× and 5000×, respectively. Such higher magnified micrographs in both Figure 3 and Figure 4 show a reasonable noticed change in the surface morphology of the studied RH samples upon exposure to various doses of γ-radiation. Specifically, the surface roughness of the samples increased with increasing γ-radiation doses up to 15 kGy, as shown in Figure 3a–d, as well as in Figure 4a–d. Meanwhile, the obtained FESEM micrographs of the RH-25 sample (Figure 3e and Figure 4e) show a smoother surface in comparison with the other studied samples, since surface roughness could be attributed to surface oxidation or the removal of organic compounds such as gases upon treatment of the samples [36]. In fact, a common effect of gamma irradiation on the surface morphology of polymers and biomass materials has been shown to be a monotonic trend that their surface roughness increases with increases in the irradiation doses [37,38,39]. Such a trend is observed by the formation of cracks on the surface as well as free radicals due to gamma irradiation [40]. On the other hand, an adverse trend of surface roughness decreasing with increasing gamma irradiation doses has also been reported [41,42]. Specifically, the surface roughness of polymer electrolytes became smoother and a decline in their spherulite structure occurred as the gamma irradiation dose increased [41]. The obtained small variations in the surface roughness of the investigated samples with changes in their γ-radiation could also be reflected in their adsorption capacity, as will be discussed later.

**Figure 5:**
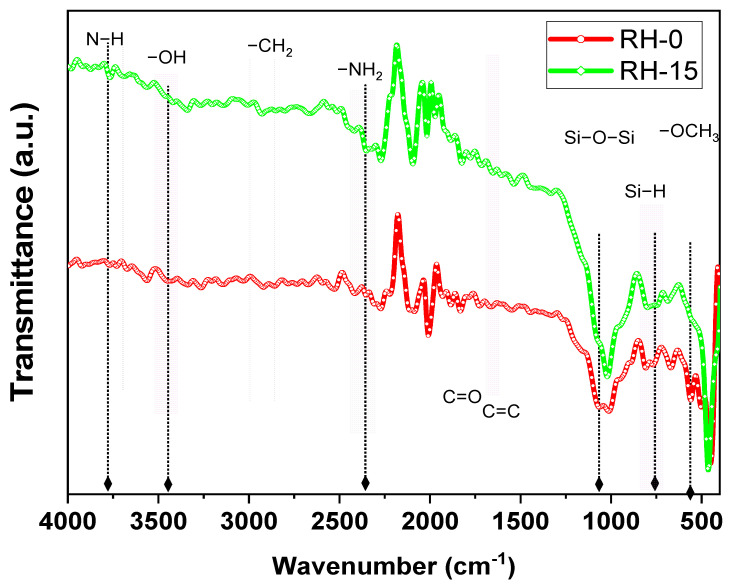
FTIR spectra for RH-0 and RH-15 samples.

Figure 5 shows the FTIR spectra for two of the rice husk samples, namely RH-0 and RH-15. The major functional groups are presented and identified on the FTIR spectra as seen in Figure 5. The hydroxyl functional group usually appears in the range of 3000–3700 cm^−1^ which in our case showed a peak at around 3448 cm^−1^. The existence of such peaks is an indication of free hydroxyl groups in both samples. This result is the same as those reported for rice husk in the literature (see for example [43]). The other functional groups were all stretching vibrations and included the following: aliphatic C–H groups, carbonyl (C=O), C=C alkenes, siloxane Si–O–Si, Si–H and alkane –OCH3 groups [44]. It is worth mentioning here that the major functional groups are highlighted in Figure 5. The peak vibrations occurring around 1064 cm^−1^, 767 cm^−1^ and 559–501 cm^−1^ corresponded to the Si–O–Si, Si–H and –OCH3 functional groups, respectively. In addition, the peak at 455 cm^−1^ occurred due to the bending of siloxane (Si–O–Si) vibrations [45]. The alkane −CH_2_ had a functional vibration appearing at about 2919 cm^−1^. The vibrations that appeared at 1720 cm^−1^ and 1666 cm^−1^ can be attributed to the carbonyl (C=O) and C=C of the alkene and aromatic groups, respectively, which were likely representative of hemicellulose and lignin [43].

For the comparison between two samples, dotted vertical lines were drawn to show the comparison between the peaks that were different between the two samples due to gamma irradiation. All the peaks at such vertical lines showed a decrease or disappearance upon the gamma irradiation of the RH. These peaks were of polar groups such as OH, Si–O–Si, Si–H and –OCH_3_. The formation of such peaks on the surface provided a significant cationic exchange with the adsorbent dye [44] and/or caused an activation of carbon’s incorporation into the structure [45]. On the other hand, peaks that included N–H and NH2 functional groups showed increases upon the gamma irradiation of the RH samples as shown in Figure 5. These results are in good agreement with the BET results as will be discussed below. 

**Figure 6:**
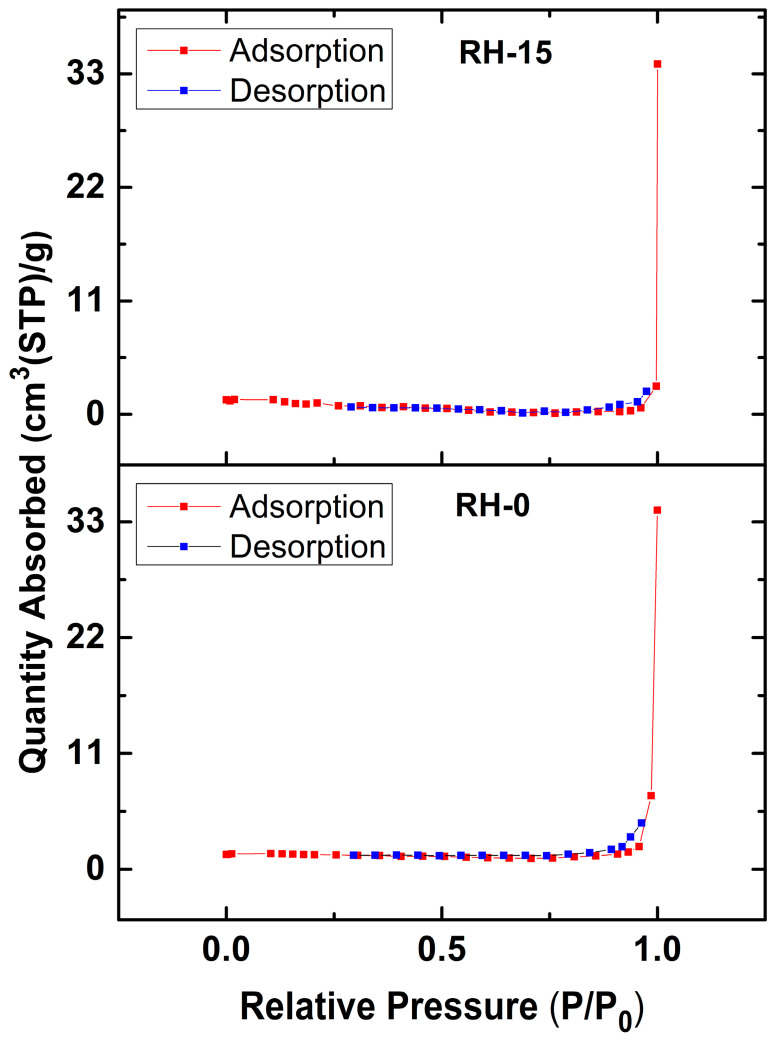
BET analysis of the nitrogen adsorption–desorption isotherms for RH-0 and RH-15.

The BET surface area, characteristic activation energy of adsorption (E_o_) and total pore volume results are tabulated in Table 1 for the RH-0 and RH-15 samples. The irradiated sample by γ-rays, RH-15, showed a lower surface area than the non-irradiated sample, RH-0, as illustrated in Table 1. This result is in agreement with the FTIR results of these two samples. However, the total pore volume value for the RH-15 sample was 16.216 × 10^−3^ cc/g, which is higher than that recorded for RH-0 (7.056 × 10^−3^ cc/g). Such behavior is in good agreement with the reported surface area and pore size analysis results for carbon cloth with and without γ-irradiation [46]. Moreover, the estimated E_o_ value decreased upon exposure to γ-irradiation, signifying an increase in the sample’s pore size. This result confirmed that gamma radiation induced pore widening and the mesopore structure. Indeed, the mesopore structure with a broad distribution of pore sizes encouraged the retention of adsorbents in the pores. Such a result was reflected in the adsorption capacity of the investigated samples as will be discussed later. Moreover, Figure 6 shows a dramatic drop in the amount of adsorbed nitrogen with decreasing gas pressure. Such behavior of the nitrogen adsorption–desorption isotherms is in good agreement with the type III adsorption isotherm [47]. So, this result confirmed the formation of microporous and mesopore structures in the investigated rice husk samples with a limited adsorption capacity [48].

### 3.2. Urolene Blue Dye Adsorption

#### 3.2.1. Determination of Equilibrium Time

**Figure 7:**
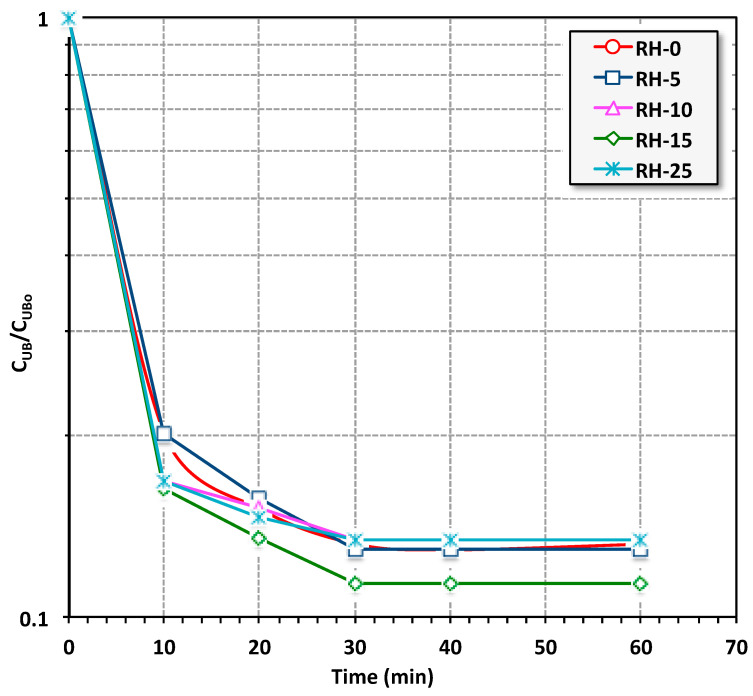
Effects of RH and UB solution contact time on UB dye removal by various RH-based adsorbents (UB = 10 ppm, RH-dose = 0.05 g/L, pH 6.6 and T = 26 °C).

For the object of evaluating the adsorption performances between the aqueous effluent loaded with Urolene Blue dye solution (10 mL of 10 ppm UB concentration at pH 6.6) and the solid adsorbent material (kept at 0.05 g/L for all RH samples), the effect of the contact time profile was included at room temperature. In order to evaluate the adsorption isotherm time, adsorption testing was performed at diverse contact times ranging from 5 to 150 min. It can be noted that all RH adsorbents that were used (i.e., RH-0, RH-5, RH-10, RH-15 and RH-25) showed an increase in UB removal with the longer applied contact time, as shown in Figure 7. The results are shown only up to 60 min since a plateau was attained after 30 min. A similar trend was elucidated for all the RH adsorbents and the isotherm time was recorded after 30 min of contact time. However, the highest removal rate overall corresponded to the RH-15 adsorbent. The increasing UB uptake with contact time could be associated with the driving force and abundance of available active adsorption sites. Initially, UB was greatly adsorbed when the adsorption concentration was high where the driving force was at its maximum and the RH surface with active adsorption sites was highly available. This means that the active sites of the RH in the initial stage of the adsorption test were unoccupied and the solute concentration was very high. However, when the time surpassed more than 30 min, the available RH sites were occupied with the molecules of the UB dye. Hence, the number of vacant sites on the RH surface regularly declined and the overall adsorption uptake was also reduced. Such a result of a decreasing contaminant uptake from an aqueous stream with the prolonging of time was previously reported by Tony [49] and Farha et al. [2] on the adsorption of dye effluents by bagasse and carbon nanotubes, respectively, as well as, Nour et al. [50], on treating phenolic materials using clay minerals. 

#### 3.2.2. Effect of Initial Loading of Urolene Blue

**Figure 8:**
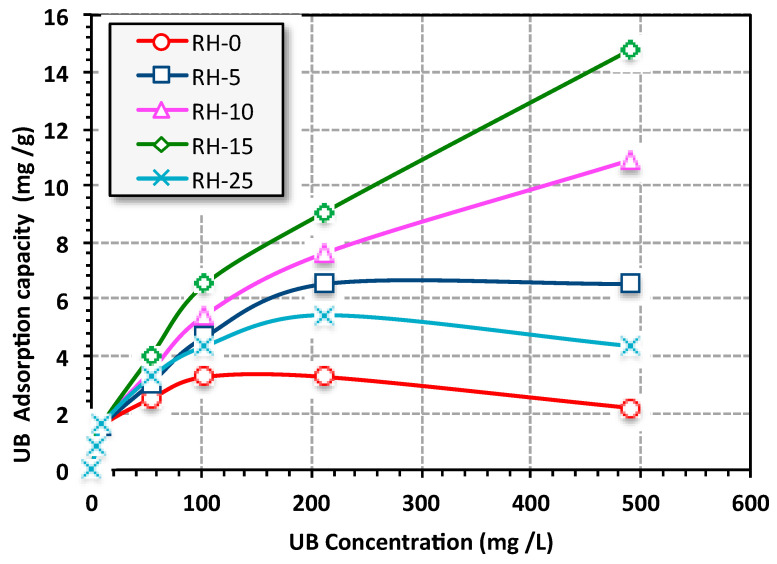
Effects of UB concentration on the adsorption capacity (isotherm adsorption time = 30 min, RH-dose = 0.05 g/L, pH 6.6 and T = 26 °C).

Figure 8 illustrates the UB dye elimination using various RH-based green adsorbents as a function of UB concentration through various RH adsorbents (RH-0, RH-5, RH-10, RH-15 and RH-25) while all the other operating parameters were kept constant (T = 26 °C, pH 6.6 and 0.05 g/L of RH-dose). The adsorption uptake that is displayed in Figure 8 exhibit elevation in the adsorption capacity as the UB increased from 5 to 500 ppm for all adsorbents over the isotherm time. The adsorption uptake increased through the initial UB load. It is also noteworthy to mention that for the adsorbent materials of RH-15 and RH-10, it can be seen that with an increase in the dye concentration from 5 to 500 ppm, the adsorption capacity increased. However, it did not reach the plateau in the studied range. Such upsurges could be associated with an enhancement in the interaction of the UB dye molecules and the RH at a higher adsorbate UB aqueous solution concentration. Such a trend associated with an increase in UB adsorbate provides the essential driving force needed to overcome the mass transfer resistance between the UB aqueous solution and the solid phase of RH [51]. Overall, the RH-15 sample presented the highest UB uptake. This could be related to the surface area available of the RH-15 sample as seen under SEM (Figure 3 and Figure 4) and in the BET results (Table 1). As a result, such a dose of γ-irradiation in this sample is efficient for surface modification. The UB adsorption capacity of RH-15 reached 14.7 mg/g in comparison to 10.88, 6.53, 4.35 and 2.17 mg/g for RH-10, RH-5, RH-25 and RH-0, respectively. The tendency of an increasing adsorption uptake from increasing the initial UB adsorbate concentration is significantly associated with the excess adsorbate molecules surrounding the RH active sites. However, the adsorption capacity was reduced for other adsorbents compared to the RH-15 adsorbent due to the disappearance of the highly adsorbent surface due to the γ-irradiation doses, which might have hindered the adsorption tendency and further affected the adsorption uptake. The available cited published literature mentioned a similar trend for methylene blue dye adsorption through various adsorbents [52]. 

#### 3.2.3. Evaluation of RH-Dose Effect on Adsorption Uptake

**Figure 9:**
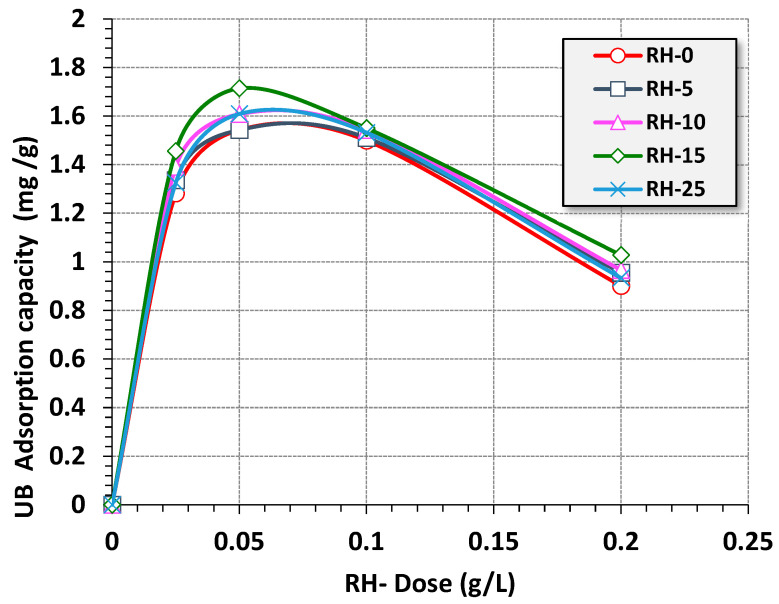
Effects of RH dose on the adsorption capacity (isotherm adsorption time = 30 min, UB concentration = 10 ppm, pH 6.6 and T = 26 °C).

The effect of different dosages of RH materials as bioadsorbents on adsorption uptake was assessed and the results are shown in Figure 9. For all RH adsorbents, the adsorbent doses increased from 0.025 to 0.2 g/L and the data revealed that an elevation in the added RH dose to the adsorption media resulted in an incremental increase in the adsorption uptake capacity. However, the maximum adsorption uptake was equivalent to 0.05 g/L for all RH-based bioadsorbents. The maximum adsorption uptake units were recorded as 1.54, 1.56, 1.61, 1.72 and 1.60 for RH-10, RH-5, RH-25 and RH-0, respectively, when the UB load (10 ppm), pH (6.6) and temperature (26 °C) were kept constant. Hence, the utilization rate of the RH adsorbent was subsequently reduced as the adsorbent dose was in a reversible relationship with the unit adsorption capabilities. In sum, the unit RH dose resulted in the aggregation and crowding of the adsorption medium. Thus, the available surface and active sites for adsorption then declined [53]. This adsorption trend and its relation to the adsorption dose was previously reported in the literature using bioadsorbents for aqueous polluted dye wastewater treatment [54]. 

#### 3.2.4. Evaluation of pH Effect on Adsorption Uptake

**Figure 10:**
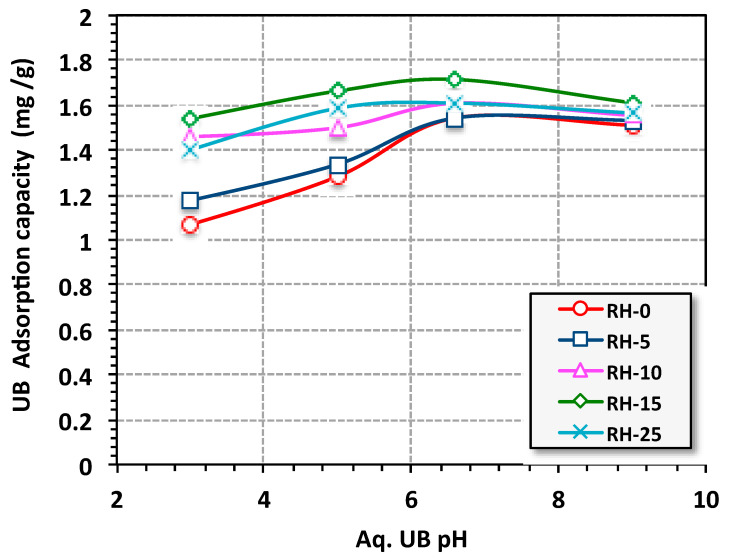
Effects of pH on the adsorption capacity (isotherm adsorption time = 30 min, UB concentration = 10 ppm, RH-dose = 0.05 g/L and T = 26 °C).

According to the literature [52,53], pH is categorized as a significant variable that affects the adsorption uptake tendency. Based on this, for the aqueous stream containing UB dye, pH was altered and the adsorbent RH dose was then added to evaluate the effect of pH variation on the RH uptake. The data displayed in Figure 10 elucidate the adsorption RH uptake capacity of UB dye at different pH values (3.0, 5.0, 6.6 and 9.0). The results in Figure 10 indicate that the RH showed different adsorption capacities by changing the pH of the solution and the dye capacity strengthened when the original pH of the solution (pH 6.6 without further adjustment) was applied. Such an investigation is associated with the point of zero charge. The point of zero charge of RH was recorded at pH 6.18, which in this regard, encompassed the repletion forces between the positive surface of the RH above the point of zero charge and the positive dye molecules. Hence, the cationic molecules of the dye were easily adsorbed on the negative acidic medium due to the electrostatic attraction between the negatively charged sorbent and positively charged UB dye in aqueous solution [55]. Lower adsorption behavior of the UB dye was recorded at low pH since at low pH, the presence of H^+^ ions is probably high and such ions are competing with the cationic groups of the UB dye at the adsorption sites [56]. Hence, the overall adsorption uptake was reduced. Moreover, the adsorption capacity was reduced at a high pH value due to the repletion forces between the positive charge on the surface of the RH adsorbent and the cationic UB dye molecules. Therefore, the cationic molecules were easily adsorbed below the point of zero charge of the RH substance. It is also essential to mention that, although the pH of 6.6. showed a maximum adsorption capacity, other pH values still showed dye removal. Moreover, on the industrial scale, the pH value of the stream could be adjusted prior to treatment. Some researchers [55,57,58] have highlighted this phenomenon, in treating various pollutants through the use of different materials. 

#### 3.2.5. Evaluation of Temperature Effect on Adsorption Uptake

**Figure 11:**
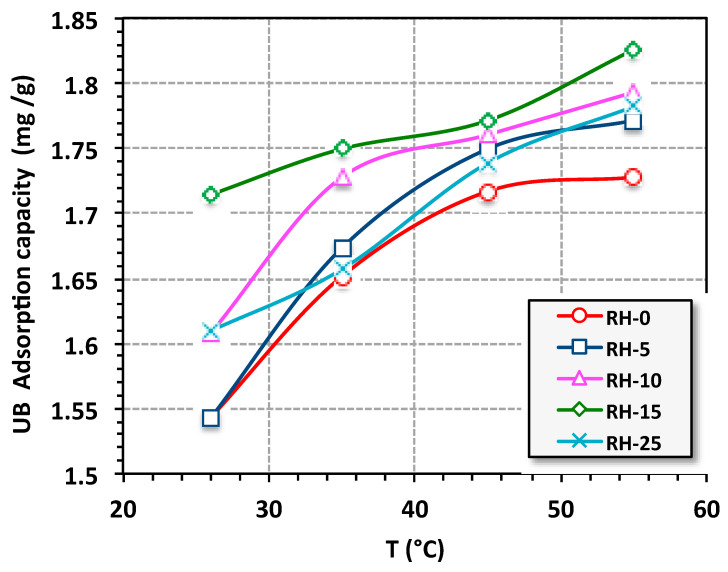
Effects of temperature on the adsorption capacity (isotherm adsorption time = 30 min, UB concentration = 10 ppm, RH-dose = 0.05 g/L and pH 6.6).

The influence of temperature on the adsorptive capacity of the various irradiated RH samples was assessed for the UB dye. The data in Figure 11 represent an enhancement in the adsorption uptake for all adsorbent samples as the temperature increased from 26 to 55 °C. Thus, dye elimination was high at a high temperature of the aqueous media. Hence, the variation in the adsorption uptake signifies the importance of temperature on the adsorption of the UB onto the RH bioadsorbent system [59]. Such adsorption uptake enhancement at a higher temperature might have been due to the endothermic nature of the adsorption process. This could be associated to the alteration of the chemical structure of the UB molecule as the temperature changed. Moreover, there were changes that appeared in the adsorption kinetics with the temperature variation. McKay et al. [60,61] previously showed the results of an investigation of the dye removal through numerous adsorbents, namely filler earth and fried clay, which was due to the attractive forces between the biomass surface and the dye molecules.

#### 3.2.6. Isotherm Modeling

**Figure 12:**
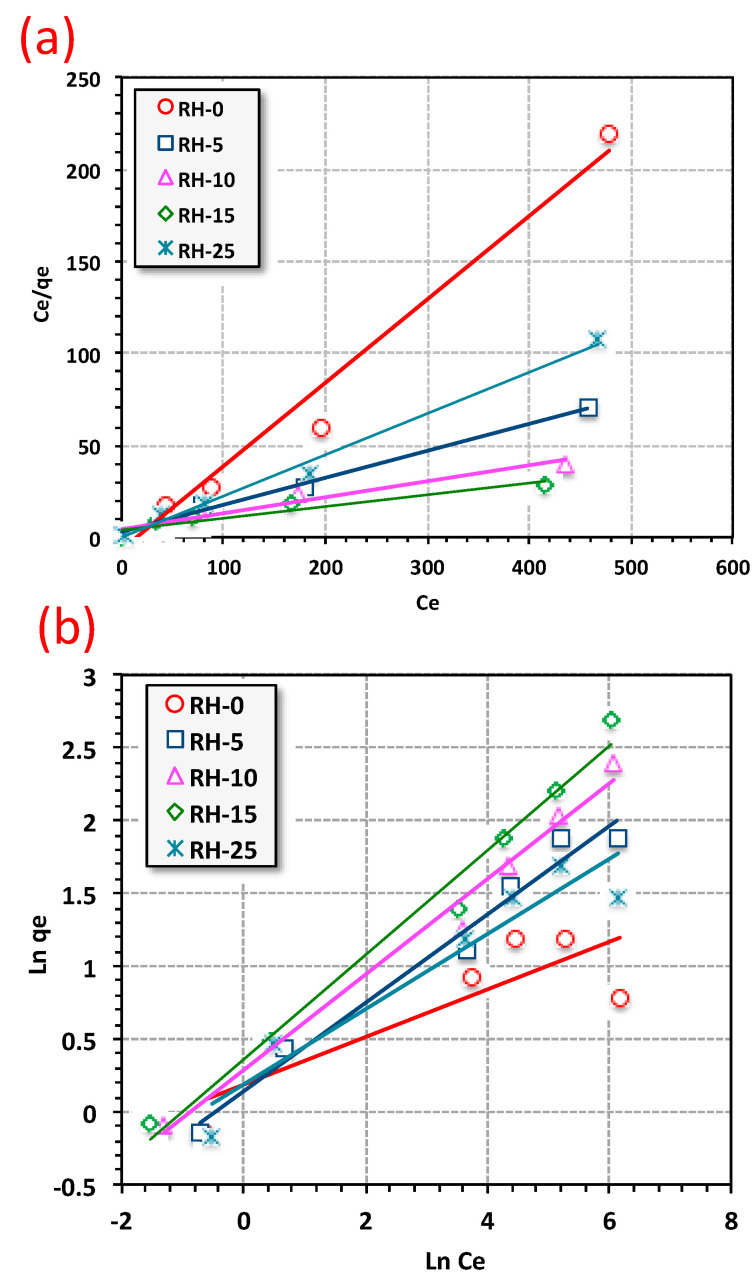
Comparison of (**a**) Langmuir and (**b**) Freundlich models of UB removal on RH adsorbent capacity (adsorption time = 30 min, adsorbent dose = 0.05 g/L, pH 6.6).

In order to lead up to real life applications, the adsorption isotherms were investigated. In this regard, a relationship between the adsorbate, Urolene Blue dye concentration in bulk and the adsorbed amount at the interface was obtained. The isotherms of adsorption were carried out at room temperature (26 °C) for the concentrations of 5 to 500 ppm of Urolene Blue dye on the five irradiated RH-based adsorbents using the most optimal experimental conditions that were mentioned above (see Figure 12 and Table 2). Although there were shortcomings in the current applied models [5], they provide an indication for estimating the type of adsorption reaction.

Langmuir and Freundlich models were used to analyze the isotherm results of the rice husk adsorption system. Based on correlation coefficient values (*R*^2^) that are shown in Table 2, the Langmuir model was the best model that could describe the data for all five adsorbents (RH-0, RH-5, RH-10, RH-15 and RH-25). The Langmuir isotherm showed the highest correlation coefficient values (0.94–0.98) in comparison to the Freundlich model (0.73–0.94) as tabulated in Table 2. Therefore, the UB adsorption process was a homogenous one. The RH adsorbents were also validated as reaching monolayer coverage until the saturation of the rice husk active sites occurred [62]. Furthermore, the heterogeneity constant (1/*n)* indicates that sorption is favorable if the *n* value is greater than unity [63]. As tabulated in Table 2, the *n* values indicate that the Urolene Blue adsorption of all the irradiated rice husk samples was signified as favorable. 

Interestingly, the data in Table 2 indicate a difference in the modes of adsorption that occurred for the various irradiated samples (RH-0, RH-5, RH-10, RH-15 and RH-25), which is likely due to the differences in their material characteristics, such as their degree of porosity due to the effect of radiation. This result could indicate that RH-15 is a superior material for water purification over other RH-based samples as it adsorbs irrespectively of the Urolene Blue characteristics.

Overall, adsorption by the rice husk for all applied samples exhibited the best affinity to the Langmuir isotherm mode. Such an investigation is in accordance with the microspore nature of the material according to the data explored by BET, meaning that only monolayer coverage can be accomplished. Not unsurprisingly, the Freundlich isotherm could not be applied to highlight the model data as a result of the nature of the rice husk adsorbent material. It is worth mentioning that based on the BET results and isotherm model analysis of the adsorption, the nature of the sample material was of a mesoporous hydrophobic material. 

#### 3.2.7. Adsorption Kinetics

The magnitude and mechanism of adsorption reactions can be elucidated by studying their reaction kinetic models [64,65,66]. From such a concept, the various applied kinetic models were evaluated to investigate the controlling mechanism of the Urolene Blue adsorption uptake from the aqueous media. However, according to [5], such models possess some inadequacy, but they can still give some indication of adsorption kinetics. In the current investigation, Lagergren’s pseudo-first- and pseudo-second-order models were assessed to describe the relevant adsorption results at 10 ppm Urolene Blue, 26 °C, pH 6.6 and 0.05 g-_RH_/L. The linear forms of the applied models are displayed in Table 3. The validity of the evaluated kinetic models was based on the assessment of their correlation coefficients. The pseudo-second-order kinetic model showed the highest correlation coefficient values. The *R*^2^ displayed a 0.99 value for all RH types compared to Lagergren’s, which indicates that the present system may have followed the pseudo-second-order model. This suggests that the reaction was dependent on adsorption capacity instead of on Urolene loading. This behavior is in agreement with the previous finding by [49] using sugarcane waste for dye adsorption. However, Ghasemi et al. [67] described their sorption reaction as following the first-order kinetic model.

A comparison of the current work with previous systems that used Urolene Blue was carried out to achieve the feasibility of the current study. The studies that are illustrated in Table 4 are based on treatments using low-cost adsorbent materials. It could be concluded that an efficient adsorption capacity was attained for such various adsorbents; however, it is noteworthy to mention that a lesser amount of irradiated rice husk was used. Using high amounts might present some disadvantages such as the need for biomass and chemical agents for the activation of such materials, which makes the procedure costly and also causes the formation of toxic chemical byproducts. In addition, the adsorption technique, which is categorized as a physicochemical treatment, results in the production of secondary pollutants. Thus, reducing the adsorbent doses could reduce the occurrence of secondary pollutants since they transfer the pollutants from one phase to another. Additionally, the current methodology activates biomass using a green methodology by γ-irradiation without using chemicals, since avoiding the use of such hazardous chemicals gives a greener adsorbent in comparison with other low-cost biomass materials. Furthermore, according to the literature [50], successive washing with distilled water or a solvent was used for dye removal which allowed for the drying of the adsorbent material in the oven to be reused, which could regenerate the adsorbent material. In this regard, the material could possibly be sustainable. Toxic dye molecules could also be recovered for further reuse. These advantages make the current technique a suitable candidate as a much better and cheaper environmentally friendly process compared to the other listed techniques in Table 4.

Rice husk is a base adsorbent for the removal of organics according to the previous cited data in the literature [76,77]. It is also noteworthy to mention that, although the current study is based on the adsorption of Urolene Blue dye as a simulated pharmaceutical dye containing wastewater using rice husk as an adsorbent material, the adsorption technique on rice husk could be used for the elimination of organics as well. Thus, the process could be suggested as an efficient method in treating real pharmaceutical effluent that is compromising several organics in wastewater streams. 

## 4. Conclusions

The current work exhibits the feasibility of rice husk and rice husk treated with γ-irradiation at various doses for Urolene Blue removal as a pharmaceutical effluent abatement from aqueous streams. SEM, BET and FTIR were used to investigate the changes in the rice husk surface as a result of various γ-irradiation doses. The surface roughness of the investigated samples displayed a non-monotonic trend when the γ-irradiation dose was varied. The surface roughness was increased up to 15 kGy and became smoother for the sample irradiated by 25 kGy. Upon γ-irradiation, surface modification changes and pore widening as well as mesopore structures were obtained. All the samples were applied to Urolene Blue and the highest adsorption capacity was recorded of 14.7 mg/g for the RH-15 sample within 30 min of adsorption time. Furthermore, the changes in the adsorption parameters as a result of the γ-irradiation dose, i.e., the aqueous effluent pH, UB concentration and temperature besides the rice husk dose were investigated. The results showed the optimal pH recorded for the mother solution as being 6.6. The Langmuir isotherm model was adequately fitted to the adsorption of Urolene Blue for all the rice husk bioadsorbents. BET and isotherm model analyses verified the nature of the obtained samples as being a hydrophobic mesoporous nature. Finally, kinetic modeling was investigated and the second-order kinetic model was successfully fitted to the adsorption data. This investigation needs further work in order to discover more real life applications. 

## Figures and Tables

**Table 1:** The surface area, characteristic energy of adsorption (E_o_) and total pore volume of RH-0 and RH-15 samples.

Sample	Surface Area (m^2^/g)	E_o_ (kJ mol^−1^)	Total Pore Volume (cc/g)
**RH-0**	0.10224	0.7488	7.056 × 10^−3^
**RH-15**	0.01862	0.4526	16.216 × 10^−3^

**Table 2:** Isotherm parameters for Urolene Blue adsorption on different γ-irradiated RH adsorbents *.

Kinetic Model	Isotherm Parameter	RH-0	RH-5	RH-10	RH-15	RH-25
**Langmuir**	*a_L_* (L/mg)	0.24	0.15	0.159	0.01	0.08
$\frac{C_{e}}{q_{e}}=\frac{1}{K_{L}}+\frac{a_{L}}{K_{L}}C_{e}$	*K_L_*	0.79	0.69	0.86	0.25	0.45
	*Q_o_* (mg/g)	3.26	4.67	5.40	15.69	5.58
	*R* ^2^	0.98	0.96	0.96	0.94	0.98
**Freundlich**	*K_F_*	6.11	3.29	3.05	2.78	3.87
$ln\left(q_{e}\right)=lnK_{F}+\frac{1}{n}lnC_{e}$	*n*	1.91	1.15	1.33	1.43	1.21
	*R* ^2^	0.73	0.94	0.93	0.94	0.91

* *C_e_*: equilibrium UB concentration (mg/L); *q_e_*: equilibrium adsorption capacity; *a_L_* and *K_L_*: Langmuir constants; *Q_o_*: monolayer adsorption capacity (mg/g); *K_F_*: Freundlich constant; *n*: heterogeneity constant.

**Table 3:** Comparing Lagergren’s pseudo-first- and pseudo-second-order models for Urolene sorption on RH systems *.

Kinetic Model	Parameter	RH-0	RH-5	RH-10	RH-15	RH-25
**Lagergren’s first-order**	*q_e_*, mg/g	0.13	0.12	0.04	0.07	0.06
$((\log\left(q_{e}-q_{t}\right)=\frac{K_{1}}{2.303}t+log(q_{e}))$,	*k*_1_, min^−1^	0.37	0.34	0.39	0.43	0.43
	*R* ^2^	0.96	0.94	0.9	0.92	0.92
**Pseudo-second-order**	*q_e_*, mg/g	1.72	1.72	1.69	1.72	1.68
($\frac{t}{q_{e}}=\frac{1}{K_{2}q_{e}^{2}}+\frac{1}{q_{e}}t$)	*k*_2_, g·mg/min	1.185	1.10	2.23	1.63	2.57
	*R* ^2^	0.99	0.99	0.99	0.99	0.99

* q_e_ and *q_t_*: amounts of UB adsorbed at equilibrium and time *t*; *t*: time; *k*_1_ and *k*_2_: Lagergren’s first- and pseudo-second-order rate constant.

**Table 4:** Comparison of different low-cost adsorbent treatment systems to the radiated rice husk.

Low-Cost Adsorbent Name	pH	Temperature	Adsorbent Dose	Initial Dye Concentration	Adsorption Capacity (mg/g)	Ref.
Radiated rice husk	pH 6.6	Room temperature	0.05 g/L	50 mg/L	14.7	**Current study**
Walnut shell	pH 8	Room temperature	0.1 g/L	200 mg/L	18	[68]
Wild carrot	pH 6	Room temperature	0.05 g/100 mL	NA	298	[69]
Coconut shell	pH 8	30 °C	0.1 g/100 mL	NA	51	[70]
Bare palm branches	NA	60 °C	5 g/L	400 mg/L	14	[51]
Corn cob	pH 5.6	30 °C	0.12 g/100 mL	NA	217	[71]
Mango peels	pH 5.6	30 °C	0.14 g/L	NA	278	[72]
Rubber leaf	pH 5.6	30 °C	0.1 g/100 mL	NA	263	[73]
Mango leaf	pH 5.6	Room temperature	NA	100 mg/L	156	[74]
Jackfruit leaf	PH 10	Room temperature	NA	200 mg/L	267	[75]

## Data Availability

All data generated or analyzed during this study are included in the published article.
